# Optimization of Difluorophenazine-Based Polymers via Substituent Modifications for Solar Energy Applications

**DOI:** 10.3390/ma19153349

**Published:** 2026-08-06

**Authors:** Puspitasari, Jaehyeong Kim, Rajalingam Agneeswari, Jae-Hoon Lee, Suhee Song, Won-Ki Lee, Wang Yong Yang, Jin Young Kim, Youngeup Jin

**Affiliations:** 1 Department of Industrial Chemistry, Pukyong National University, Busan 48513, Republic of Korea; 2 School of Energy and Chemical Engineering, Ulsan National Institute of Science and Technology (UNIST), Ulsan 44919, Republic of Korea; kimjjae0@unist.ac.kr (J.K.);; 3 BB21+ Project Team, Pukyong National University, Busan 48513, Republic of Korea; 4 Department of Polymer Engineering, Pukyong National University, Busan 48513, Republic of Korea; 5 Department of Chemistry, University of Tennessee at Chattanooga, Chattanooga, TN 37403, USA

**Keywords:** organic solar cells, benzodithiophene, phenazine, conjugated polymers, donor substituent engineering

## Abstract

A series of donor–acceptor π-conjugated polymers based on Difluorophenazine acceptor and benzodithiophene donor units with different substituents, hydrogen (H), chlorine (Cl), fluorine (F), and sulfur (S), was designed and synthesized to investigate how donor-side substituent modification influences optoelectronic properties. The substituent variation systematically modulated the optical band gaps (1.72–1.82 eV) and HOMO energy levels (−5.42 to −5.58 eV). When applied in bulk heterojunction solar cells with the Y6 acceptor, these polymers delivered power conversion efficiencies ranging from 3.85% to 7.79%. The fluorinated polymer P(BDTTF-TffPzT) exhibited the best performance, with *J*_sc_ = 19.65 mA cm^−2^, *V*_oc_ = 0.825 V, and FF = 0.481. These findings establish that donor-side substituent engineering is an effective molecular design strategy for tuning optoelectronic properties and device performance in phenazine-based polymer systems.

## 1. Introduction

Organic solar cells (OSCs) have attracted increasing attention as a promising photovoltaic technology because of their lightweight nature, mechanical flexibility, and potential for low-cost solution processing. Continuous advances in material design and device engineering have led to remarkable improvements in OSC performance over the past decade [1,2]. According to the National Renewable Energy Laboratory (NREL) Best Research-Cell Efficiency Chart and the latest Solar Cell Efficiency Tables, certified single-junction OSCs have achieved PCEs exceeding 19–20%, underscoring the rapid progress enabled by the development of advanced donor and acceptor materials together with optimized device architectures [3,4].

Beyond organic photovoltaic materials, inorganic semiconductors have also been extensively explored in solar energy conversion owing to their superior operational stability and well-established charge transport properties [5,6,7]. Recent comprehensive reviews highlight the important roles of binary oxide ceramics such as TiO_2_, ZnO, Al_2_O_3_, SiO_2_, CeO_2_, Fe_2_O_3_, and WO_3_ in photovoltaic applications, emphasizing their versatility as functional components in solar cell architectures [8]. In parallel, significant progress in solution-processed organic photovoltaic systems has been summarized in the recent literature, demonstrating the continued advancement of high-efficiency organic solar cells through materials innovation and processing strategies. Importantly, this intrinsic molecular design flexibility in organic semiconductors enables precise tuning of donor–acceptor interactions, which is critical for optimizing exciton dissociation and charge transport in photovoltaic systems [9]. In addition, efficient light absorption remains a fundamental requirement for high-performance organic solar cells because the conversion of solar energy begins with the interaction between incident photons and photoactive materials [9,10]. As a result, molecular design strategies that can modulate the optical absorption characteristics of conjugated semiconductors are of particular importance for enhancing photovoltaic performance [10].

This molecular design flexibility is particularly important in non-fullerene acceptor (NFA)-based systems, where subtle structural variations in both donor and acceptor components can significantly influence optical properties, energy level alignment, and overall photovoltaic behavior [11]. NFAs have fundamentally reshaped the OSC landscape by offering broader absorption, tunable energy levels, and reduced non-radiative recombination losses [12,13]. Polymer donor systems and high-performance NFA-based material platforms have demonstrated PCEs exceeding 19% through rational molecular design strategies that minimize energetic disorder and enhance interfacial charge transfer [14,15]. Moreover, multicomponent and ternary active-layer strategies have further expanded the efficiency frontier, with recent reports approaching or surpassing 20% PCE by simultaneously optimizing morphology control and charge transport pathways [16,17]. For example, Fu et al. [14] demonstrated a record 19.31% efficiency in binary OSCs through crystallization regulation, while Li et al. [15] achieved over 20% efficiency by developing a high-crystallinity NFA with high photoluminescence quantum yield. Xiao et al. [16] reviewed recent strategies for achieving efficiencies beyond 19%, while Wu et al. [17] demonstrated that active layer reconstruction can enable 19.46–efficient all-polymer organic solar cells.

From a materials perspective, the outstanding performance of modern OSCs arises from the synergistic optimization of donor and acceptor components within the bulk heterojunction (BHJ) architecture [9]. Previous studies have shown that tailored conjugated polymers featuring broad absorption characteristics and appropriately aligned frontier energy levels can contribute to improved photovoltaic performance in organic solar cells [11,12]. In parallel, morphology regulation strategies including additive engineering, solvent selection, and interfacial layer design have also been widely employed to optimize active-layer processing and device performance in bulk heterojunction systems [13,18]. These findings emphasize that both molecular structure and solid-state organization must be carefully controlled to realize high photovoltaic performance [9].

Among the various donor building blocks explored to date, benzodithiophene (BDT) has emerged as one of the most widely adopted cores because of its strong electron-donating character, high planarity, and structural versatility. BDT-based donor polymers have served as key components in many high-efficiency OSC systems, where subtle molecular modifications of the donor unit play an important role in tuning optical absorption characteristics and frontier molecular orbital energy levels [19,20]. Recent studies further demonstrate that small variations in substituent chemistry can significantly influence optical and electronic properties, thereby affecting device performance [21]. Phenazine is an attractive electron-deficient building block owing to its strong aromaticity, excellent chemical stability, and tunability [22]. Its rigid and highly planar conjugated structure facilitates efficient intermolecular π–π stacking, which is beneficial for charge transport and molecular packing [23]. Although phenazine-based materials have demonstrated excellent photovoltaic performance as electron acceptors (up to 17.7%) [23] and guest donors (up to 17.3%) [24], their application as electron donor building blocks in conjugated polymers remains largely unexplored, highlighting the need for systematic investigation. Building on this concept, halogenation and heteroatom substitution have become powerful molecular design strategies for regulating the electronic structure and intermolecular interactions of conjugated polymers. Halogenation is an effective strategy for tuning the optical absorption, energy levels, and molecular packing of conjugated materials [22]. In particular, fluorine incorporation has been widely reported to modulate frontier energy levels and optical properties in conjugated polymer systems [25,26]. Incorporating alkylthiol substituents can significantly influence the physicochemical properties, active-layer micro-morphology, and photovoltaic performance of organic solar cells [27].

Moreover, recent studies have demonstrated that precise morphology control can enable equally high efficiencies in organic solar cells processed from different solvents [28], while advanced molecular and interfacial engineering strategies have pushed device efficiencies from over 20% in single-junction OSCs to 26% in perovskite/organic tandem solar cells [29]. This knowledge gap is especially evident for polymer donors incorporating fluorinated phenazine acceptor segments, where substituent engineering represents a potentially powerful yet underexplored strategy for regulating optoelectronic properties and morphology. Most previous studies focused on achieving high efficiency through global optimization, without systematically comparing substituent effects. This work fills that gap by comparing H, Cl, F, and S substituents on the donor unit while keeping the polymer backbone and molecular weight consistent. Therefore, a systematic investigation of substituent effects in benzodithiophene–phenazine copolymer systems is required to clarify how different functional groups modulate optoelectronic properties and photovoltaic performance. Benzodithiophene–phenazine-based conjugated polymer systems provide an ideal platform to systematically explore how donor-unit substituent variations influence the optoelectronic properties and device behavior in non-fullerene acceptor-based organic solar cells.

In this work, we report the design and synthesis of a series of π-conjugated polymers constructed from difluorophenazine acceptor motifs and structurally differentiated benzodithiophene donor units. The polymer backbones incorporating unsubstituted, chlorine-, fluorine-, and sulfur-functionalized benzodithiophene segments were prepared via Stille cross-coupling polymerization. Through a systematic comparative study within this chemically consistent polymer platform, the effect of donor-side substituent variation on optical absorption, frontier energy levels, morphological characteristics, and photovoltaic performance was evaluated. This work aims to elucidate how donor-unit substituent modification governs structure-property-performance relationships in difluorophenazine-based conjugated polymer systems for organic photovoltaic applications.

## 2. Materials and Methods

### 2.1. Materials

All reagents and solvents were purchased from commercial suppliers (Sigma-Aldrich (St. Louis, MO, USA), Tokyo Chemical Industry (TCI) (Tokyo, Japan), and Alfa Aesar (Ward Hill, MA, USA)) and used without further purification unless otherwise stated. All reactions were performed under a nitrogen atmosphere.

### 2.2. Polymer Synthesis

The polymers P(BDTTH-TffPzT), P(BDTTCl-TffPzT), P(BDTTF-TffPzT), and P(BDTTS-TffPzT) were prepared via Stille cross-coupling polymerization following the procedure described in the Supporting Information. A flame-dried flask containing the respective monomers (BDTTH, BDTTCl, BDTTF, or BDTTS and TffPzT) in chlorobenzene was purged with argon for 30 min. Subsequently, Pd_2_(dba)_3_ and P(o-tol)_3_ were added, and the mixture was heated at reflux for 36 h under argon. After cooling to room temperature, the solution was precipitated into methanol. The resulting polymer was purified by Soxhlet extraction with methanol and acetone. Detailed synthetic procedures are provided in the Supporting Information.

### 2.3. Characterization

Gel permeation chromatography (GPC) measurements were performed using a Waters GPC system equipped with a refractive index detector (2414, Waters Corporation, Milford, MA, USA). o-Dichlorobenzene (o-DCB) was used as the eluent at 140 °C with a flow rate of 1.0 mL min^−1^. The system was calibrated using polystyrene standards. UV–Vis absorption spectra were recorded using a UV–Vis spectrophotometer (Cary 5000, Agilent Technologies, Santa Clara, CA, USA) in both solution and thin-film states. Polymer solutions were prepared in chlorobenzene (1 wt%), while thin films were fabricated by spin-coating onto quartz substrates at 1000–1500 rpm. Cyclic voltammetry (CV) measurements were performed using an Ivium electrochemical workstation in a 0.1 M tetrabutylammonium hexafluorophosphate (Bu_4_NPF_6_) acetonitrile solution. A glassy carbon working electrode, platinum wire counter electrode, and Ag/AgCl reference electrode were used. Ferrocene/ferrocenium (Fc/Fc^+^) was used as the internal standard. Polymer thin films were deposited onto the working electrode prior to measurement. Atomic force microscopy (AFM) images were obtained using a Bruker Dimension Icon system (Bruker Corporation, Billerica, MA, USA) operated in tapping mode to investigate the surface morphology of polymer:Y6 blend films prepared from a chloroform solution with a donor:Y6 weight ratio of 1:1.2, followed by thermal annealing at 100 °C for 5 min. The surface morphology was recorded over a scan area of 2 × 2 μm^2^. ^1^H and ^13^C NMR spectra were recorded using a Bruker Avance 400 MHz spectrometer (Bruker Corporation, Billerica, MA, USA) at room temperature. Chemical shifts (δ) are reported in ppm relative to tetramethylsilane (TMS) as internal reference. Deuterated chloroform (CDCl_3_) was used as the solvent.

### 2.4. Device Fabrication and Measurement

Inverted-type organic solar cells with the configuration ITO/PEDOT:PSS/active layer/PDINN/Ag were fabricated. The active layer was spin-coated from a chloroform solution containing the polymer donor and Y6 acceptor at a 1:1.2 weight ratio. Various spin-coating rates (2000 and 3000 rpm) and thermal annealing conditions (100 °C for 5 min) were employed for the fabrication of the films. A 150 nm thick Ag electrode was deposited by thermal evaporation through a shadow mask. J–V characteristics were measured under simulated AM 1.5G illumination (100 mW cm^−2^) using a Keithley 2400 source meter (Keithley Instruments, Solon, OH, USA). EQE spectra were recorded using a calibrated monochromator system.

## 3. Results and Discussion

**Figure 1:**
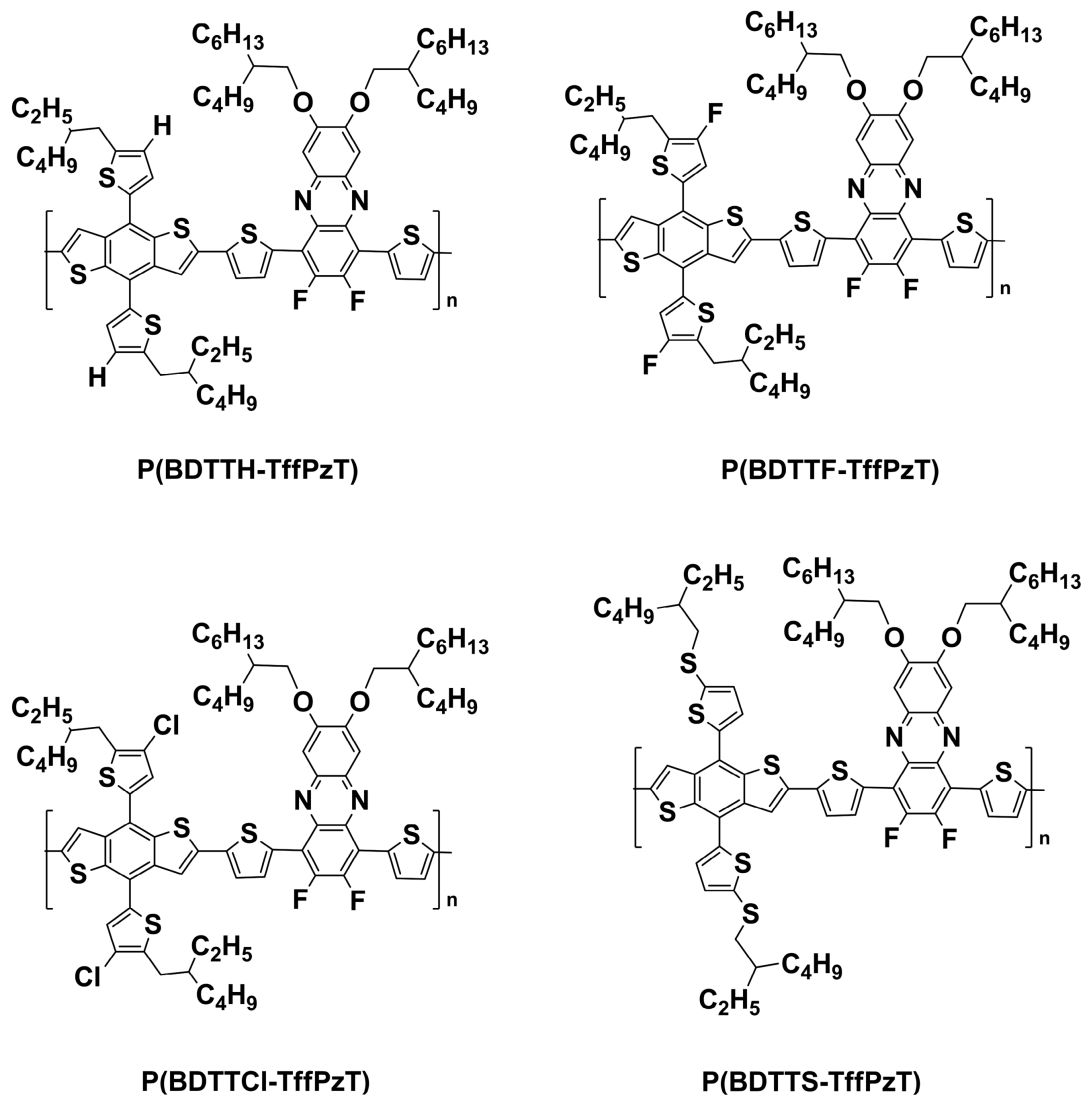
Structural differences in P(BDTTH-TffPzT), P(BDTTCl-TffPzT), P(BDTTF-TffPzT), and P(BDTTS-TffPzT).

**Scheme 1:**
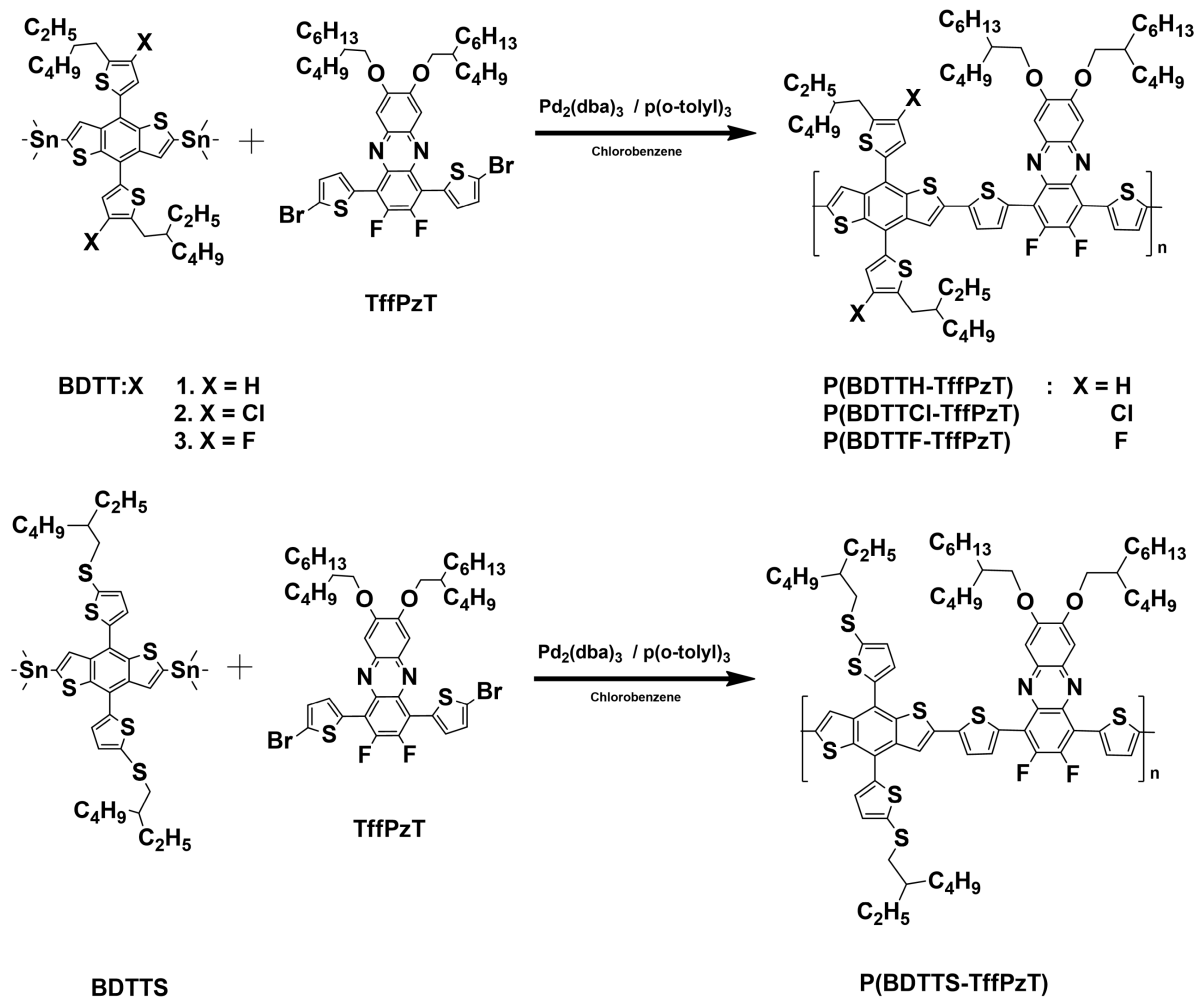
Synthetic routes of P(BDTTH-TffPzT), P(BDTTCl-TffPzT), P(BDTTF-TffPzT), and P(BDTTS-TffPzT).

The polymer series was prepared according to the synthetic routes outlined in Scheme 1, with detailed experimental procedures described in the Supporting Information (SI). Dibrominated Difluorophenazine was employed as a key electron-accepting unit, while a series of benzodithiophene (BDT) derivatives (BDTTH, BDTTCl, BDTTF, and BDTTS) were used as electron-donating comonomers. Through a Stille-based cross-coupling polymerization process, the target π-conjugated polymers P(BDTTH-TffPzT), P(BDTTCl-TffPzT), P(BDTTF-TffPzT), and P(BDTTS-TffPzT) (Figure 1) were successfully obtained.

For clarity, although the polymer naming includes the TffPzT unit, the acceptor framework remains structurally consistent across the series, incorporating a fixed acceptor motif throughout all polymers. Therefore, the present study focuses on isolating donor-side substituent effects rather than variations in the acceptor structure. By maintaining a consistent acceptor framework and overall backbone architecture, the observed variations in optoelectronic behavior can be primarily attributed to the electronic and steric characteristics of the substituents on the BDT donor segment. The number-average molecular weights (Mn) ranged from 27.6 to 34.7 kDa with relatively narrow dispersities (Đ = 2.15–2.23), suggesting that the observed variations in device performance primarily originate from substituent effects rather than differences in molecular weight (Table 1).

### 3.1. Optical and Electrochemical Characteristics

**Figure 2:**
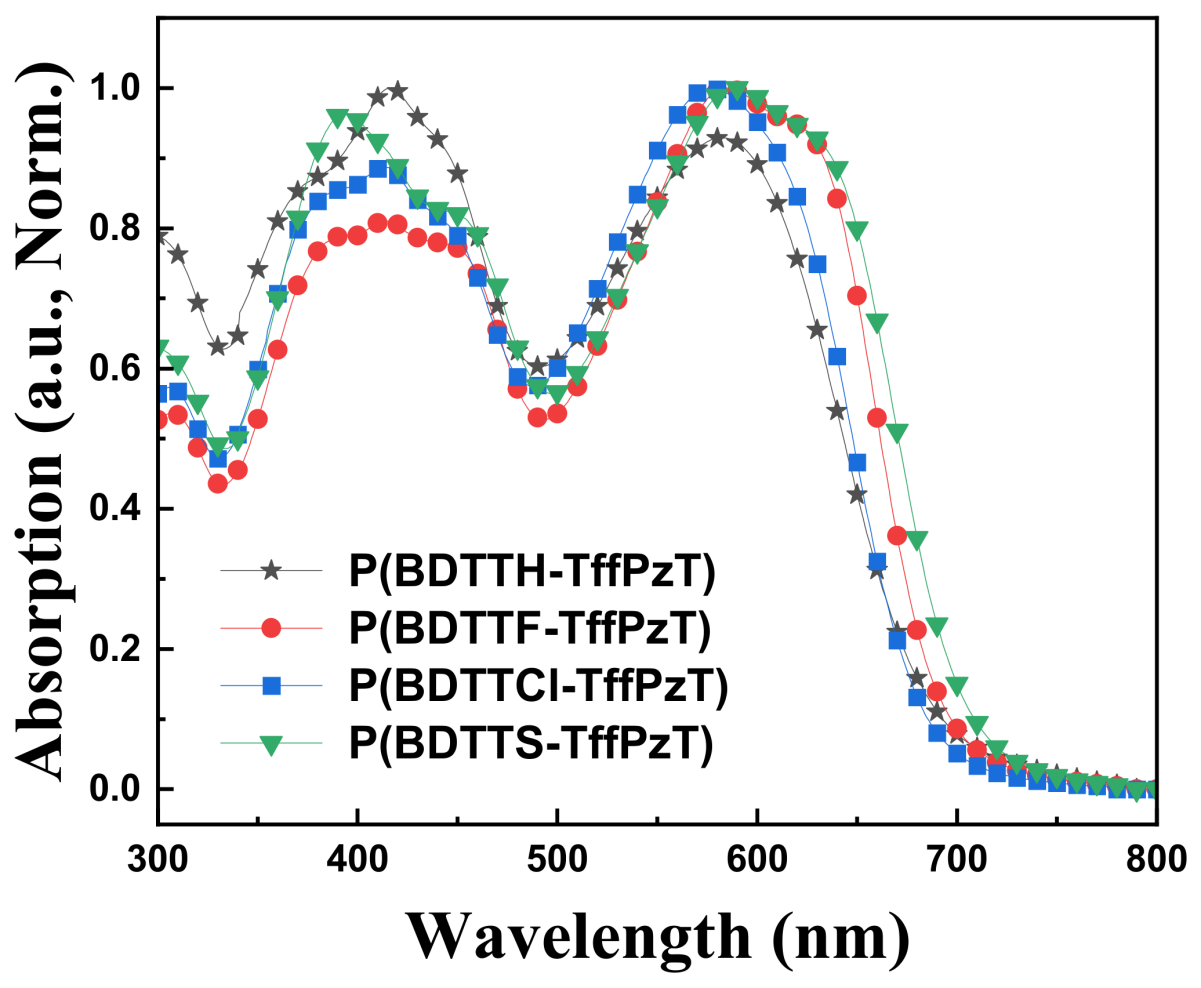
UV–visible absorption spectra in a thin film formed via spin-cast from a solution in dichlorobenzene (1 wt%).

To elucidate the influence of donor substituent variation on the optical behavior of the polymers, thin-film UV–visible absorption spectra were analyzed (Figure 2), with the key optical parameters compiled in Table 2. Across the series, the polymers exhibit similarly broad absorption features extending from approximately 300 to 800 nm, consistent with those typically observed in donor-acceptor conjugated backbones. The absorption profiles comprise a higher-energy component arising from π–π* transitions along the conjugated backbone and a lower-energy component associated with donor-acceptor charge-transfer characteristics. P(BDTTH-TffPzT) and P(BDTTCl-TffPzT) display similar absorption edge positions at 705 and 706 nm, corresponding to an optical energy gap of approximately 1.82 and 1.81 eV, respectively. Despite the comparable onset wavelengths, clear differences emerge in both spectral shape and absorption intensity. P(BDTTH-TffPzT) and P(BDTTCl-TffPzT) show similar absorption edges at 715 and 745 nm, with optical band gaps around 1.79 and 1.72 eV, respectively. The main difference is in the spectral shape, where P(BDTTH-TffPzT) exhibits a weaker low-energy absorption tail than the fluorinated derivatives. This observation is consistent with previous reports showing that fluorine substitution enhances intramolecular charge transfer and extends absorption toward lower photon energies [30,31]. In contrast, the chlorine-substituted polymer exhibits a sharper spectral profile, which is consistent with the electronic influence of chlorine substitution on the polymer backbone, potentially introducing subtle electronic modulation. This behavior is attributed primarily to electronic modulation rather than steric effects, in line with previous studies reporting minimal steric influence of chlorine substitution in similar conjugated systems. Furthermore, introduction of fluorine substituents in P(BDTTF-TffPzT) leads to a noticeable bathochromic shift in the absorption edge to 715 nm, yielding a reduced optical bandgap of 1.79 eV. The red shift observed with fluorine substitution is consistent with earlier reports on fluorinated donor-acceptor polymers [25,26]. The most pronounced red shift is observed for P(BDTTS-TffPzT), which exhibits an absorption edge at 745 nm and the smallest optical bandgap of 1.72 eV. This result reflects the strong influence of sulfur substitution on the electronic structure of the polymer, resulting in a more pronounced low-energy absorption profile.

**Figure 3:**
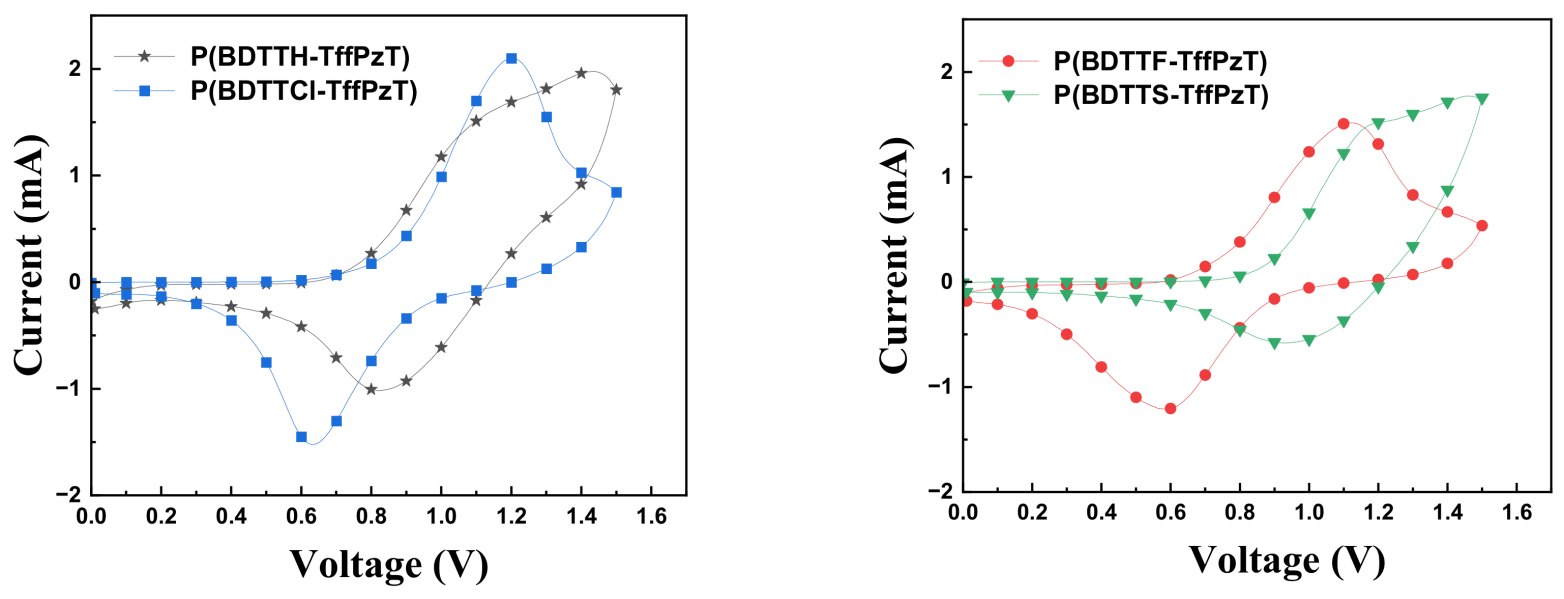
Cyclic voltammetry curves of P(BDTTH-TffPzT), P(BDTTCl-TffPzT), P(BDTTF-TffPzT), and P(BDTTS-TffPzT).

To further clarify substituent-dependent electronic effects, the electronic band structures of polymers were systematically evaluated using cyclic voltammetry. Representative voltammograms are illustrated in Figure 3, while the quantitative electronic descriptors are summarized in Table 3 to facilitate systematic comparison. All polymers exhibit well-defined oxidation onsets, enabling reliable estimation of their HOMO energy levels.

P(BDTTH-TffPzT) displays an onset oxidation potential of 0.62 V, corresponding to a frontier valence orbital positioned at −5.42 eV. Upon chlorine substitution, P(BDTTCl-TffPzT) shows a pronounced shift in oxidation onset to 0.65 V, leading to a HOMO position at −5.45 eV. This trend is consistent with the electron-withdrawing character of chlorine, which stabilizes the electronic structure of the conjugated backbone. Among the polymer series investigated in this study, P(BDTTF-TffPzT) exhibits the most stabilized frontier orbitals, featuring its frontier orbital energies stabilized at −5.44 eV for the valence band edge (HOMO) and −3.65 eV for the conduction band edge (LUMO). The observed lowering of both energy levels is consistent with the electron-withdrawing nature of fluorine substitution, which may contribute to the corresponding device-performance trend. In contrast, P(BDTTS-TffPzT) displays relatively shallower electronic states, with its frontier valence positioned at −5.58 eV and conduction band edges at −3.86 eV. The sulfur-substituted polymer exhibits relatively higher-lying energy levels compared to the halogen-substituted systems, indicating a distinct electronic influence associated with sulfur substitution within the donor unit. Overall, the slight differences between the values discussed and those listed in Table 3 originate from variations in onset determination and numerical precision.

### 3.2. Photovoltaic Characteristics

**Figure 4:**
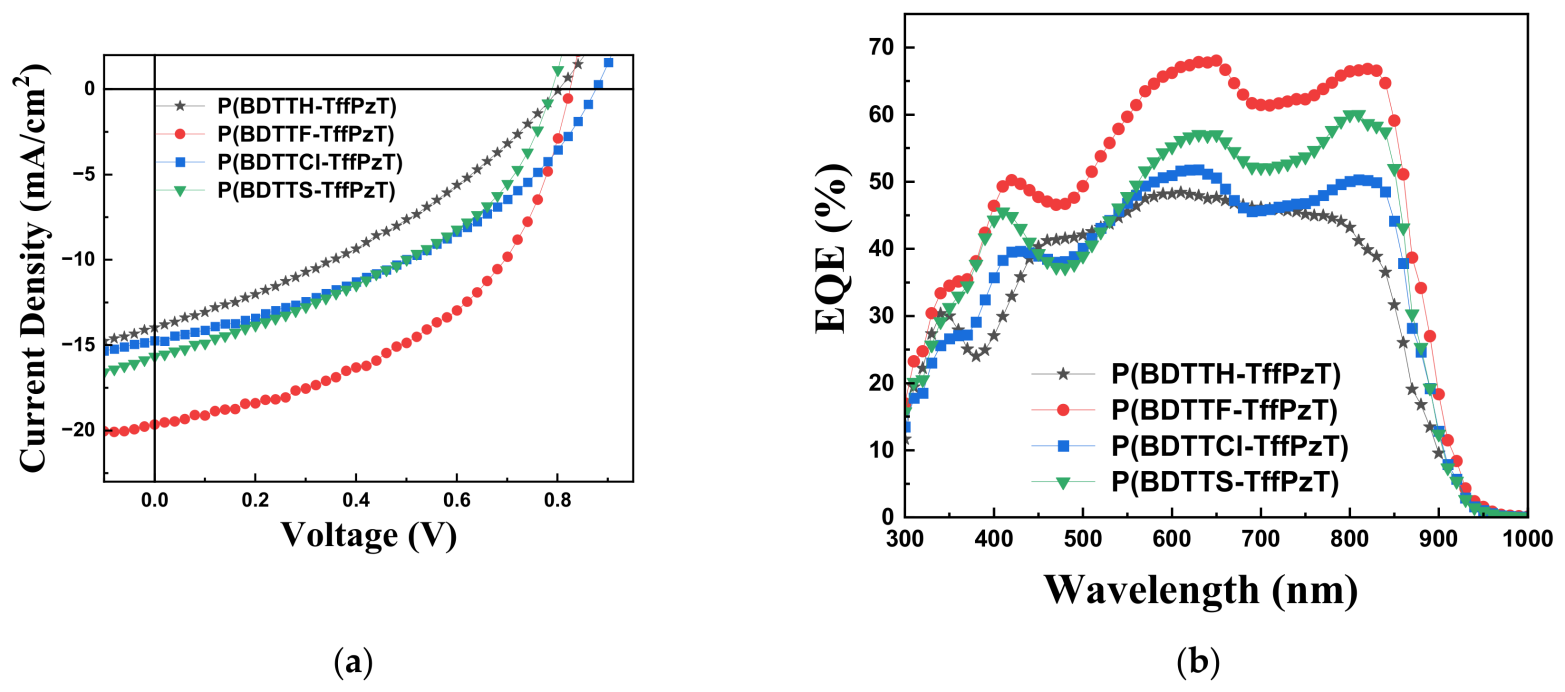
(**a**) J-V characteristics of devices based on P(BDTTH-TffPzT), P(BDTTCl-TffPzT), P(BDTTF-TffPzT), and P(BDTTS-TffPzT) under 100 mW/cm^2^ AM 1.5G illumination. (**b**) EQE spectrum.

To assess the impact of substituent-induced optoelectronic modulation at the device level, the polymers were employed as donor components in bulk heterojunction photovoltaic devices. The corresponding current-voltage characteristics and EQE spectra are shown in Figure 4. Different spin-coating rates (2000 and 3000 rpm) and thermal annealing conditions (100°C, 5 min) were applied in Table 4. Device performance exhibits a clear dependence on donor-side substituent identity. Devices based on P(BDTTH-TffPzT) exhibit relatively modest performance, with a maximum power conversion efficiency (PCE) of 3.85%, accompanied by a short-circuit current density (*J*_sc_) of 13.97 mA cm^−2^, an open-circuit voltage (*V*_oc_) of 0.803 V, and a fill factor (FF) of 0.343 under annealed conditions. The relatively lower device performance is consistent with its comparatively narrower absorption profile and less stabilized frontier energy levels within the investigated polymer series. Upon incorporation of chlorine substituents, P(BDTTCl-TffPzT) shows improved performance, delivering a maximum PCE of 5.11%, with a *J*_sc_ of 14.76 mA cm^−2^, a *V*_oc_ of 0.876 V, and an FF of 0.395 under optimized conditions (3000 rpm, annealed).

Among the polymer series, P(BDTTF-TffPzT) delivers the highest efficiency, achieving a maximum PCE of 7.79%, with a *J*_sc_ of 19.65 mA cm^−2^, a *V*_oc_ of 0.825 V, and an FF of 0.481. The higher performance of the fluorinated polymer is consistent with trends seen in other fluorinated donor polymers [11,25]. This trend is generally consistent with the corresponding optical and electrochemical characteristics observed for the fluorinated polymer system. In contrast, devices based on P(BDTTS-TffPzT) exhibit moderate performance, with a maximum PCE of 5.06%, a *J*_sc_ of 15.66 mA cm^−2^, a *V*_oc_ of 0.789 V, and an FF of 0.409. Although P(BDTTS-TffPzT) exhibited the narrowest optical bandgap and the most red-shifted absorption profile within the investigated polymer series, it did not produce the highest photovoltaic performance. Despite its broader absorption characteristics, P(BDTTS-TffPzT) exhibited lower *J*_sc_, *V*_oc_, and FF values than P(BDTTF-TffPzT). The lower *V*_oc_ observed for P(BDTTS-TffPzT) is consistent with its comparatively shallower HOMO energy level, while the lower FF suggests that differences in active-layer film characteristics may also contribute to the observed device behavior.

The EQE spectra follow trends similar to the J-V results. The best-performing P(BDTTF-TffPzT) device exhibits a broad and high photo-response, and the integrated current density obtained from the EQE spectrum (~19.2 mA cm^−2^) agrees well with the J-V result (19.65 mA cm^−2^), confirming measurement consistency. A similar structure-property relationship was observed in our previously reported phenazine-based polymer system [32], highlighting the value of systematic structure–property investigations within chemically consistent polymer series. The fluorinated derivative exhibited the most balanced photovoltaic characteristics, suggesting that fluorine substitution on the donor unit effectively modulates the electronic properties of the conjugated backbone under the investigated device conditions.

### 3.3. Morphology Characteristics

**Figure 5:**
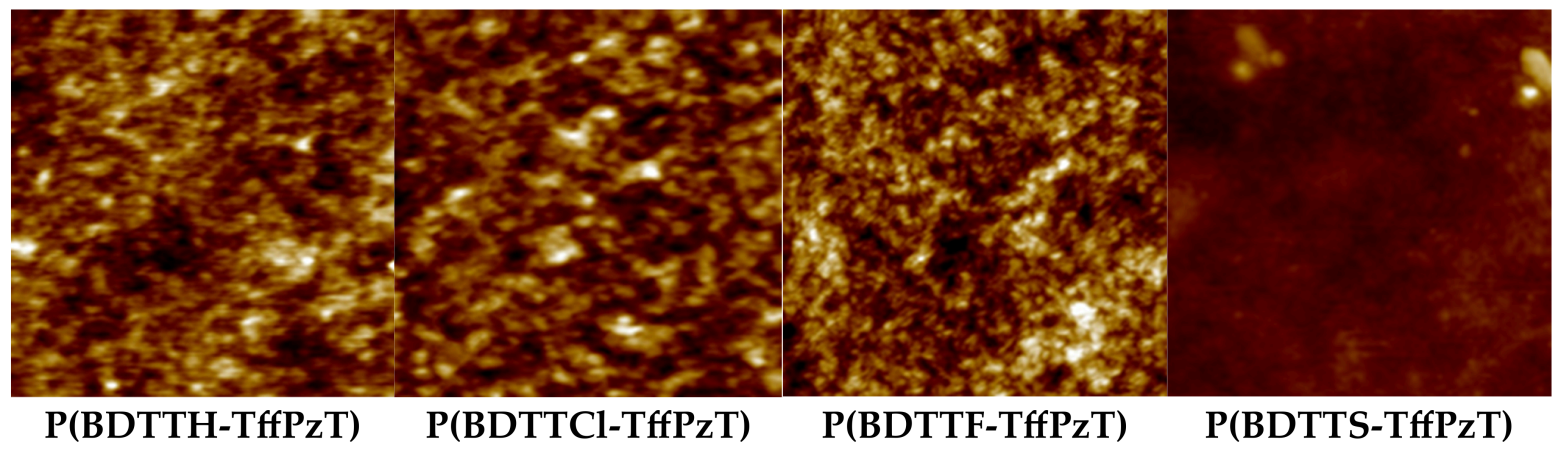
AFM topography images of polymer:Y6 blend films based on P(BDTTH-TffPzT), P(BDTTCl-TffPzT), P(BDTTF-TffPzT), and P(BDTTS-TffPzT). The corresponding RMS roughness values are 1.05, 1.60, 1.66, and 3.39 nm, respectively.

To further investigate the surface characteristics of the polymer blend films, the nanoscale morphology of the polymer blend films was investigated by atomic force microscopy (AFM) in order to evaluate the influence of donor-side substituent variation on the surface characteristics of the polymer:Y6 blend films, as shown in Figure 5. The blend films were prepared from polymer:Y6 solutions with a donor:Y6 weight ratio of 1:1.2 in chloroform without additional additives, followed by spin coating and thermal annealing at 100 °C for 5 min under the same fabrication conditions used for photovoltaic device preparation. The blend films based on P(BDTTH-TffPzT), P(BDTTF-TffPzT) and P(BDTTCl-TffPzT) displayed relatively uniform nanoscale surface textures throughout the scanned area. In contrast, the P(BDTTS-TffPzT) blend films exhibited more distinguishable nanoscale surface textural variations. Among the investigated polymers, P(BDTTS-TffPzT) displayed a less homogeneous surface morphology than the other polymer blend films. The corresponding root-mean-square (RMS) roughness values were 1.05, 1.60, 1.66, and 3.39 nm for P(BDTTH-TffPzT), P(BDTTCl-TffPzT), P(BDTTF-TffPzT), and P(BDTTS-TffPzT), respectively.

These morphological differences may partially contribute to the observed variations in photovoltaic behavior among the polymer systems. Although P(BDTTS-TffPzT) exhibited the narrowest optical bandgap (1.75 eV) and the most red-shifted absorption profile among the investigated polymer series, it did not yield the highest photovoltaic performance. This observation indicates that minimizing the optical bandgap does not necessarily correspond to optimal device efficiency in polymer solar cells. In general, a narrower optical bandgap can extend the absorption window and improve solar photon harvesting, thereby potentially enhancing photocurrent generation. However, overall device efficiency is determined by the combined balance of multiple photovoltaic parameters. Consequently, improvement in an individual photovoltaic parameter does not inherently result in superior overall device performance. Despite its extended absorption profile, P(BDTTS-TffPzT) did not exhibit the highest *J*_sc_ within the investigated polymer series, showing a lower *J*_sc_ value (15.66 mA cm^−2^) than P(BDTTF-TffPzT) (19.65 mA cm^−2^). This observation suggests that spectral broadening was not the sole determinant governing photocurrent generation in the present system. In addition, P(BDTTS-TffPzT) showed a lower *V*_oc_ (0.789 V) relative to P(BDTTF-TffPzT) (0.825 V), which is consistent with its comparatively shallower HOMO energy level (−5.36 eV for P(BDTTS-TffPzT) versus −5.54 eV for P(BDTTF-TffPzT)). Furthermore, the lower FF value of P(BDTTS-TffPzT) (0.409) compared with P(BDTTF-TffPzT) (0.481) suggests that additional factors, including differences in film characteristics, may also contribute to the observed variation in device performance. Similar morphology-dependent tendencies were also observed in our previously reported phenazine-based polymer system [32], where variations in backbone composition and phenazine side-chain architecture significantly influenced nanoscale film organization and blend-film homogeneity.

## 4. Conclusions

In this study, a series of difluorophenazine-based conjugated polymers incorporating benzodithiophene donor units with systematically varied substituents were successfully designed and synthesized via Stille cross-coupling polymerization. By maintaining a chemically consistent donor-acceptor framework while introducing hydrogen, chlorine, fluorine, and sulfur substituents on the donor unit, the influence of donor-side substituent modification on polymer optoelectronic properties and photovoltaic behavior was systematically investigated.

The results demonstrate that donor-side substituent identity significantly affects optical absorption characteristics, frontier energy levels, and device performance. Among the investigated polymer series, fluorine substitution produced the most favorable balance of optical and electronic properties, enabling P(BDTTF-TffPzT) to achieve the highest photovoltaic performance with a maximum power conversion efficiency of 7.79%. In contrast, although sulfur substitution led to the narrowest optical bandgap and most red-shifted absorption profile, this did not directly translate into superior device efficiency, indicating that optical bandgap reduction alone is insufficient for optimal photovoltaic performance. Overall, fluorine substitution on the donor unit effectively modulated the electronic properties of the conjugated backbone and produced the most balanced photovoltaic characteristics under the investigated device conditions. This work provides useful molecular design insights for the future development of difluorophenazine-based polymer donors for organic photovoltaic applications.

## Figures and Tables

**Table 1:** GPC results of the synthesized polymers.

Polymers	Mn (kDa)	Mw (kDa)	Dispersity (Đ)
P(BDTTH-TffPzT)	31.2	68.4	2.19
P(BDTTCl-TffPzT)	34.7	74.5	2.15
P(BDTTF-TffPzT)	29.8	66.3	2.23
P(BDTTS-TffPzT)	27.6	61.1	2.21

**Table 2:** Optical Properties of Polymers.

Polymer	In Solution (nm)	In Thin Film (nm)
P(BDTTH-TffPzT)	652	688
P(BDTTCl-TffPzT)	653	687
P(BDTTF-TffPzT)	688	697
P(BDTTS-TffPzT)	696	710

**Table 3:** Electrochemical Properties of the Polymers.

Polymers	Optical Band Gap ^a^ (eV)	HOMO ^b^ (eV)	LUMO ^c^ (eV)	*E*_ox_ ^d^ (V)
P(BDTTH-TffPzT)	1.82	−5.42	−3.60	0.62
P(BDTTCl-TffPzT)	1.81	−5.45	−3.64	0.65
P(BDTTF-TffPzT)	1.79	−5.44	−3.65	0.64
P(BDTTS-TffPzT)	1.72	−5.58	−3.86	0.78

^a^ Optical energy band gap was estimated from the onset wavelength of the optical absorption. ^b^ Calculated from the oxidation potentials. ^c^ Calculated from the optical band gap and HOMO energy levels. ^d^ Onset oxidation potential measured by cyclic voltammetry.

**Table 4:** Photovoltaic Performance of Solar Cells Based on P(BDTTH-TffPzT), P(BDTTCl-TffPzT), P(BDTTF-TffPzT), and P(BDTTS-TffPzT).

Polymers	Spin Rate (rpm)	Thermal Annealing (100 °C, 5 min)	*J*_sc_ (mA/cm^2^)	*V*_oc_ (V)	FF	PCE (%)
P(BDTTH-TffPzT)	2000	X	11.57	0.799	0.337	3.12
	3000	X	12.57	0.776	0.339	3.31
	2000	O	13.97	0.803	0.343	3.85
P(BDTTCl-TffPzT)	2000	X	14.22	0.880	0.347	4.35
	3000	X	13.51	0.884	0.377	4.50
	2000	O	15.61	0.874	0.374	5.10
	3000	O	14.76	0.876	0.395	5.11
P(BDTTF-TffPzT)	2000	X	20.23	0.824	0.424	7.07
	3000	X	18.80	0.827	0.470	7.31
	2000	O	20.46	0.825	0.433	7.31
	3000	O	19.65	0.825	0.481	7.79
P(BDTTS-TffPzT)	2000	X	13.94	0.788	0.382	4.20
	3000	X	15.66	0.789	0.409	5.06
	2000	O	14.63	0.770	0.393	4.43

## Data Availability

The original contributions presented in this study are included in the article. Further inquiries can be directed to the corresponding authors.

## Supplementary Materials

The following supporting information can be downloaded at: https://www.mdpi.com/article/10.3390/ma19153349/s1.
