# Supplementary material for: Optimization of Difluorophenazine-Based Polymers via Substituent Modifications for Solar Energy Applications
Source: Materials (Basel). 2026 Aug 6;19(15):3349. doi: 10.3390/ma19153349 (PMC13468687; doi:10.3390/ma19153349)
Supplement: Supplementary file 1 [file materials-19-03349-s001.zip › materials-4398276-supplementary.pdf]

Supporting Information (SI) for:

Optimization of Difluorophenazine-Based Polymers via Substituent Modifications for Solar Energy Applications.

Puspitasari[1,#], Jaehyeong Kim[2,#], Rajalingam Agneeswari[1,#], Jae-Hoon Lee[1,5], Suhee Song,[2] Won-Ki Lee[3,5], Wang Yong Yang[4], Jin Young Kim[2,*], and Youngeup Jin[1,5,*]

[1]Department of Industrial Chemistry, Pukyong National University, Busan 48513, Korea

[2]Department of Energy Engineering, Ulsan National Institute of Science and Technology (UNIST), Ulsan 44919, South Korea

[3]Department of Polymer Engineering, Pukyong National University, Busan, Korea

[4]Department of Chemistry, University of Tennessee, Chattanooga, USA

[5]BB21+ Project Team, Pukyong National University, Busan 48513, Korea

Corresponding author: yjin@pknu.ac.kr (Youngeup Jin)

Synthesis of 1,4-bis(5-bromothiophen-2-yl)-7,8-bis(2-butyloctyloxy)-2,3-difluorophenazine (TffPzT)

To a solution of 7,8-bis(2-butyloctyloxy)-2,3-difluoro-1,4-di(thiophen-2-yl)phenazine (1.5g, 2.008 mmol) was dissolved in chloroform (30ml). Then, N-bromosuccinimide (0.786g, 4.418 mmol) was added one portion. The reaction mixture was stirred at room temperature for 12hr. Then, the reaction mixture was extracted with chloroform (100ml × 3). The organic layer was dried over anhydrous $MgSO_4$. After all solvent evaporated, additional purification carried out on silica column with petroleum hexane/CH_3Cl (5:1) to give red solid of compound **TffPzT** (1.708g, 94%). ($CDCl_3$, 600 MHz, δ): 7.65 (d, 2H, J = 4.05Hz), 7.10 (d, 2H, 4.56Hz), 7.00 (s, 2H), 4.12 (t, 4H, J = 12.60Hz), 1.95 (m, 4H, J = 21.66Hz), 1.58 – 1.23 (m, 32H, J = 210.06Hz), 0.87(t, 6H, J = 14.10Hz).

Polymerization

Synthesis of **P(BDTTH-TffPzT) and P(BDTTCl-TffPzT)**

A flame dried three neck round bottom flask containing a solution of respective monomers, **BDTTH (1)** (0.23 g, 0.25 mmol) and **TffPzT** (0.23 g, 0.25 mmol) for **P(BDTTH-TffPzT)**; and **BDTTCl (2)** (0.24 g, 0.25 mmol) and **TffPzT** (0.23 g, 0.25 mmol) for **P(BDTTCl-TffPzT)**, in chlorobenzene (30 mL) was purged well with argon for 30 min. Subsequently, Pd2dba3 (14 mg) and P(o-tol)3 (28 mg) were added and the entire mixture was heated under reflux in argon for 36 h. The solution was cooled to RT and the solution was added drop wise to methanol (250 mL) with constant stirring. The precipitated polymer was allowed to settle. The precipitates were filtered and washed with methanol (50 mL) and acetone (50 mL). The polymer was then subjected to Soxhlet extraction with methanol and acetone for 24 h. The resulting solid was dried under vacuum to afford pure polymer. Yield for **P(BDTTH-TffPzT)**: 0.32 g (97%) and Yield for **P(BDTTCl-TffPzT)**: 0.33 g (95%).

Synthesis of **P(BDTTF-TffPzT) and P(BDTTS-TffPzT)**

A flame dried three neck round bottom flask containing a solution of respective monomers, **BDTTF (3)** (0.24 g, 0.25 mmol) and **TffPzT** (0.23 g, 0.25 mmol) for **P(BDTTF-TffPzT)**; and **BDTTS** (0.24 g, 0.25 mmol) and **TffPzT** (0.23 g, 0.25 mmol) for **P(BDTTS-TffPzT)**, in chlorobenzene (30 mL) was purged well with argon for 30 min. Subsequently, Pd2dba3 (14 mg) and P(o-tol)3 (28 mg) were added and the entire mixture was heated under reflux in argon for 36 h. The solution was cooled to RT and the solution was added drop wise to methanol (250 mL) with constant stirring. The precipitated polymer was allowed to settle. The precipitates were filtered and washed with methanol (50 mL) and acetone (50 mL). The polymer was then subjected to

Soxhlet extraction with methanol and acetone for 24 h. The resulting solid was dried under vacuum to afford pure polymer. Yield for P(BDTTF-TffPzT): 0.33 g (97%) and Yield for P(BDTTS-TffPzT): 0.33 g (97%).

Fabrication and analysis of polymer solar cells

To fabricate inverted type PSCs with configuration of indium tin oxide (ITO)/PEDOT:PSS/active layer/PDINN/Ag, a Ag film, 150-nm-thick, was initially deposited on an ITO surface by the sol-gel process. The solution of PDINN precursors was prepared by dissolving PDINN (1.5 mg) in methanol (1 mL). The active layer was fabricated using a chloroform with 1:1.2 ratio between solution of the polymeric donor and Y6 acceptor by spin-coating. Finally, an Ag layer, 150 nm-thick, were consecutively deposited by thermal evaporation through a shadow mask with a device area. The conditions of solar simulation were calibrated prior to the measurements by using a silicon reference cell with a KG5 filter certified by the National Institute of Advanced Industrial Science and Technology.

Donor	Spin rate [RPM]	Thermal annealing (100°C, 5min)	Jsc [mA/cm2]	Cal. Jsc [mA/cm2]	Voc [V]	FF	PCE [%]
P(BDTTH-TfFPzT)	2000	X	11.57 (11.38±0.22)		0.799 (0.795±0.002)	0.337 (0.333±0.003)	3.12 (3.01±0.08)
	3000	X	12.57 (12.00±0.44)		0.776 (0.776±0.001)	0.339 (0.340±0.001)	3.31 (3.16±0.11)
	**2000**	**O**	**13.97 (13.54±0.36)**	13.8	**0.803 (0.802±0.001)**	**0.343 (0.342±0.003)**	**3.85 (3.71±0.13)**
P(BDTTCl-TfFPzT)	2000	X	14.22 (13.65±0.40)		0.880 (0.885±0.004)	0.347 (0.348±0.007)	4.35 (4.21±0.14)
	3000	X	13.51 (12.88±0.46)		0.884 (0.888±0.004)	0.377 (0.374±0.003)	4.50 (4.27±0.16)
	2000	O	15.61 (15.12±0.34)	14.93	0.874 (0.879±0.005)	0.374 (0.374±0.003)	5.10 (4.97±0.09)
	**3000**	**O**	**14.74 (14.26±0.45)**		**0.876 (0.875±0.005)**	**0.395 (0.396±0.001)**	**5.11 (4.94±0.13)**
P(BDTTF-TfFPzT)	2000	X	20.23 (19.77±0.33)		0.824 (0.829±0.003)	0.424 (0.425±0.006)	7.07 (6.96±0.10)
	3000	X	18.80 (18.44±0.25)		0.827 (0.818±0.020)	0.470 (0.444±0.042)	7.31 (6.71±0.83)
	2000	O	20.46 (19.93±0.53)	19.57	0.825 (0.818±0.006)	0.433 (0.433±0.012)	7.31 (7.06±0.18)
	**3000**	**O**	**19.65 (19.00±0.52)**		**0.825 (0.816±0.017)**	**0.481 (0.456±0.043)**	**7.79 (7.09±0.92)**
P(BDTTS-TfFPzT)	2000	X	13.94 (13.92±0.12)		0.788 (0.783±0.003)	0.382 (0.382±0.004)	4.20 (4.16±0.03)
	**3000**	**X**	**15.66 (15.19±0.33)**	16.47	**0.789 (0.791±0.003)**	**0.409 (0.406±0.004)**	**5.06 (4.88±0.13)**
	2000	O	14.63 (14.45±0.16)		0.770 (0.773±0.004)	0.393 (0.395±0.002)	4.43 (4.41±0.02)

Table S1. Photovoltaic Performance of Solar Cells Based on P(BDTTH-TffPzT), P(BDTTCl-TffPzT), P(BDTTF-TffPzT), and P(BDTTS-TffPzT).
